# Encapsulation and adhesion of nanoparticles as a potential biomarker for TNBC cells metastatic propensity

**DOI:** 10.1038/s41598-023-33540-1

**Published:** 2023-07-29

**Authors:** Yulia Merkher, Elizaveta Kontareva, Elizaveta Bogdan, Konstantin Achkasov, Ksenia Maximova, Joshua M. Grolman, Sergey Leonov

**Affiliations:** 1 Laboratory of Innovative Medicine and Agrobiotechnology, Moscow Institute of Physics and Technology (MIPT), Dolgoprudny, Moscow Region Russia; 2 The Biomechanic Materials Lab, Technion Israel Institute of Technology, Haifa, Israel

**Keywords:** Biomarkers, Endocytosis, Breast cancer, Metastasis, Cellular imaging, Cytoskeleton, Image processing

## Abstract

Metastasis is the main cause of cancer-related mortality; therefore, the ability to predict its propensity can remarkably affect survival rate. Metastasis development is predicted nowadays by lymph-node status, tumor size, histopathology, and genetic testing. However, all these methods may have inaccuracies, and some require weeks to complete. Identifying novel prognostic markers will open an essential source for risk prediction, possibly guiding to elevated patient treatment by personalized strategies. Cancer cell invasion is a critical step in metastasis. The cytoskeletal mechanisms used by metastatic cells for the invasion process are very similar to the utilization of actin cytoskeleton in the endocytosis process. In the current study, the adhesion and encapsulation efficiency of low-cost carboxylate-modified fluorescent nanoparticles by breast cancer cells with high (HM) and low metastatic potential (LM) have been evaluated; benign cells were used as control. Using high-content fluorescence imaging and analysis, we have revealed (within a short time of 1 h), that efficiency of nanoparticles adherence and encapsulation is sufficiently higher in HM cells compared to LM cells, while benign cells are not encapsulating or adhering the particles during experiment time at all. We have utilized custom-made automatic image analysis algorithms to find quantitative co-localization (Pearson’s coefficients) of the nanoparticles with the imaged cells. The method proposed here is straightforward; it does not require especial equipment or expensive materials nor complicated cell manipulations, it may be potentially applicable for various cells, including patient-derived cells. Effortless and quantitative determination of the metastatic likelihood has the potential to be performed using patient-specific biopsy/surgery sample, which will directly influence the choice of protocols for cancer patient’s treatment and, as a result, increase their life expectancy.

## Introduction

Today cancer is the third cause of death worldwide^1^. There are different methods to detect cancer, such as blood tests with cancer-specific markers, imaging (including MRI, CT, X-ray, ultrasound techniques), and endoscopy. However, the cause of over 90% of all cancer-related deaths is metastasis^2,3^. The prediction of metastasis is challenging: the main predictors for metastasis are lymph node status, histological grade, and tumor size. Those predictors are, however, not infallible. For example, about 30% of breast cancer patients with negative lymph node status will develop metastases^4^; contrarily, histologically graded anaplastic prostatic adenocarcinoma tumors may have low metastatic potential^5^. The TNM classification, based on the size of the primary tumor and metastasis to lymph nodes or distant sites, does not consider the organs of possible metastatic growth^6^. Genetic testing, an additional technique for metastatic cancer prognosis, allows the identification of specific subtypes within the overall disease category based on differences in gene expression^7^. Unfortunately, this technique can only provide information on specifically identified genes, while in many specific cancers^6^ and cancer mutations, prognostic markers are yet undetermined. In practice, the sensitivity and specificity of individual markers may vary widely, and several physiological and pathological factors, like environmental conditions, smoking, obesity etc., can affect the results^8^. Regular histological examination of the tumor sample can take weeks and months. Uncertainty about the final diagnosis is associated with substantial biochemical distress, which affects immune defense and healing^9^. In the light of all the limitations listed above, new innovative approaches are required to accurately estimate the propensity for metastasis, preferably with rapidly provided quantitative results, independent of user bias. Breast cancer (BC) is the most commonly diagnosed cancer among women, and the second leading cause of cancer-related mortality worldwide^10^. Triple negative breast cancer (TNBC) accounts about 20% of all breast carcinomas, compared with HR-positive BCs, TNBC has a worse prognosis^11^. High malignancy, extensive heterogeneity, and drug resistance make TNBC the most appropriate candidate for developing novel prognostic methods, independent of genetic or molecular profiles^12^.

Metastatic migration and invasion through tissue are critical steps in formation of metastases. The widely used measure of cancer aggressiveness—is cell invasiveness, or the ability of a cell to invade its surroundings. Several studies have examined the mechanisms of cancer cells invasion or migration in 2-D and 3-D environments, while intracellular mechanics play an essential role in the process^13,14^. Changes in the intracellular mechanics during malignant transformation influence the invasion of cancer cells^15^. Various techniques like atomic force microscopy, subcellular laser ablation, magnetic twisting cytometry, magnetic endosome micro-rheology, and multiple particles tracking micro rheology have been employed in recent years to characterize the properties of cancer cells during intracellular processes^16–19^. The more dynamic internal environment (due to softer cell membrane, cytoplasm and nucleus) of highly metastatic (HM) cells and their sparser cytoskeleton compared to both low metastatic (LM) potential and benign cells^17,18^ make the HM cells also more capable to internalization substances from their surroundings^16^. The excellent recovery ability and plasticity of highly invasive metastatic cells are related to the actomyosin contractile apparatus and actin remodeling^20^. Actin cytoskeleton remodeling mediated by the Rho GTPases is an essential mechanism for metastatic cell invasion. During the invasion, cells apply forces through the actomyosin network^21^, which pushes the cell membrane; an increase in actomyosin-generated forces is directly correlated with increased invasiveness of metastatic cells^22^. Actin also plays an essential role in endocytosis—actin participates at multiple stages of endocytosis, including membrane invagination, scission, and propulsion of the endocytic vesicle^23^. Actively remodeling actin network cells can successfully facilitate endocytosis^24^. The drugs such as latrunculin A inhibit endocytosis in mammalian cells^25^ and, at the same time, play a major role in inhibiting cell invasion, as was tested by gel-invasion assay^26^. Therefore, there is a direct connection between the ability of cancer cells to migrate and invade and their adhesive and encapsulating propensity.

Depending on the particle size and type, internalization way and mechanism, there are three general modes of cellular endocytosis that coexist in the cell and act in parallel: phagocytosis (internalization of large solid particles > 0.5 μm), pinocytosis (0.5–5 μm vesicles) and receptor-mediated endocytosis (internalization of small < 0.5 μm particles via specific receptors). A role for endocytosis was proposed in tumor progression due to the similarity of signals and processes directing cell movement and endocytosis. Endocytosis occurs at regions of the plasma membrane involved in spreading, therefore can directly contribute to the cell migration^27^. It seems that derailed (enhanced or distorted) endocytosis can make significant contributions to sustained proliferation of cancer cells, enhanced invasiveness, and avoidance of apoptosis^28^. The multicomponent process of endocytosis affects and regulates receptor internalization, recycling and degradation, as well as cytoskeleton dynamics; this believed to alter different steps of metastasis development. The most studied endocytic mode in cancer relation is receptor-mediated endocytosis^29^, in particular, clathrin-mediated endocytosis (CME) signaling is critical in cancer and metastasis. Clathrin is upregulated explicitly in non-small-cell lung cancer (NSCLC) cells and is associated with poor prognosis^30^. The regulation of specific receptors is known to affect cancer and metastasis by clathrin- and non-clathrin-mediated internalization pathways. Caveolae-mediated endocytosis (CavME) is vital in transcytotic trafficking across cells and mechanosensing^31^. Components of CavME have a vital role in cell migration, invasion, and metastasis. Knockdown of CAV-1 in breast and prostate cancer cells reduced the velocity, directionality, and persistency of cellular migration^32^. Receptor-mediated pathways suppress cancer cell blebbing and invasion through GTPase-activating protein GRAF1^33^. The previous research indicates, that the size of (ligand-devoid) particles can determine the entry pathway. The upper size limit of endocytic pits for clathrin-mediated endocytosis is around 200 nm, and kinetic parameters may determine the almost complete internalization of such particles along this pathway rather than via caveolae. Therefore, we have chosen to concentrate our research on particles of 100-200 nm diameter (smaller particles needed special equipment to expand the detection limits for the experiments). Nanoparticle size and shape are critical physicochemical properties that determine the extent and efficiency of initial nanoparticle-cell interactions. Endocytosis-mediated studies explored various particles sizes and complex shapes with different complicated and expensive treatments and surface modifications^34^. Carboxylated polystyrene nanoparticles chosen in our study have been used previously to determine the endocytosis pathway within cancer cells, albeit this process has not been connected to the cell invasiveness yet^16^. The particles remain mainly in the cell's rim, presumably due to interactions with negatively charged plasma membrane proteins on the cell surface^35^. Here, we evaluate the short-time internalization of 100 and 200-nm diameter particles, comparing benign, LM and HM epithelial breast cancer cells^36^, and correlating the internalization efficiency to the invasive abilities of cancer cells.

## Results

**Figure 1:**
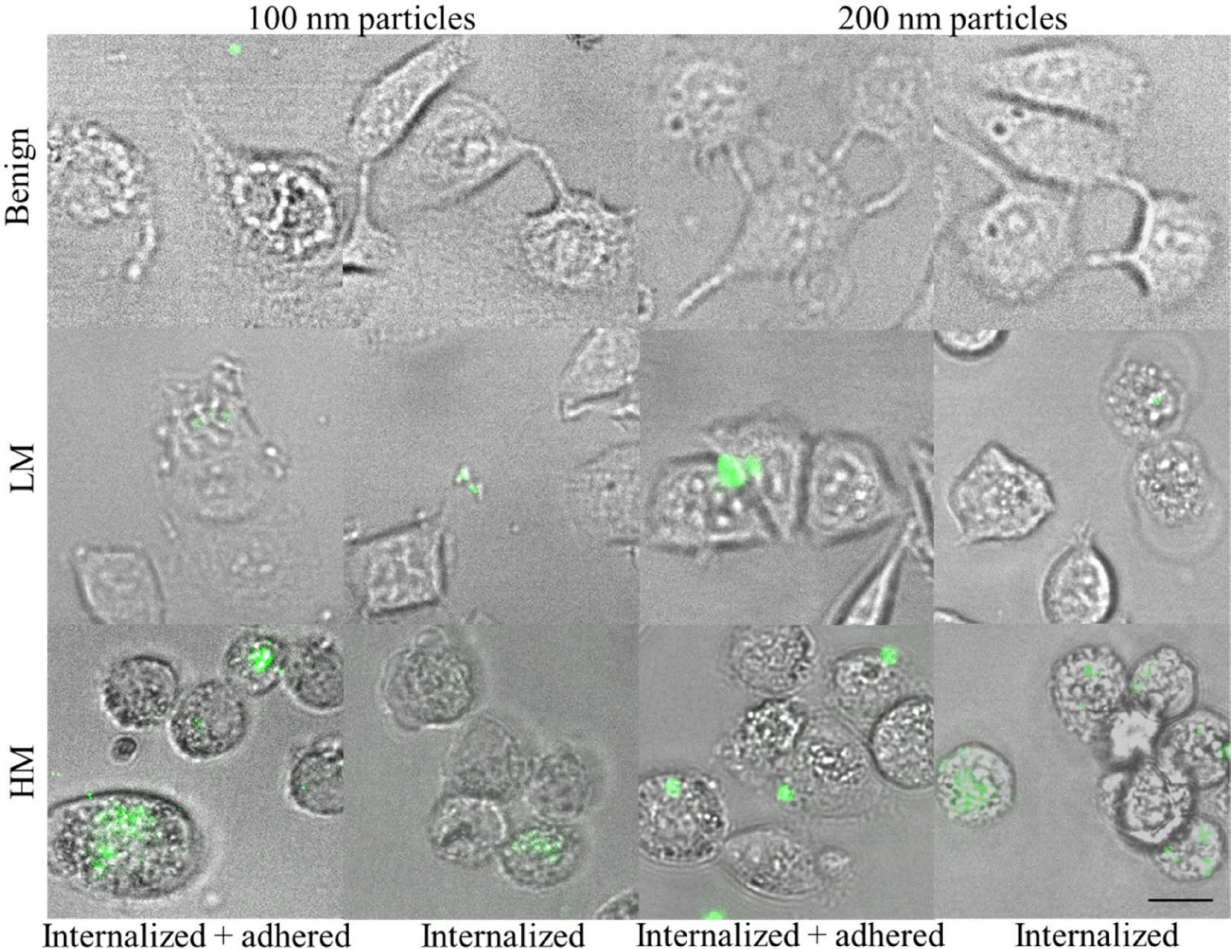
A typical images (selected cells × 5 magnification from Supplementary 1) of internalized and internalized and adhered carboxylate modified 100 nm and 200 nm nanoparticles by benign cells, cells with low (LM) and high (HM) metastatic potential. HM and LM cells were extensively washed with Trypsin or PBS after 1 h incubation with fluorescent nanoparticles, resulting in the presence of internalized or internalized and adhered beads, respectively. The scale bar is 10 µm.

Nanoparticle uptake is considered as a two-step process, where the nanoparticles initially adhere to the cell membrane and subsequently are internalized by the cells. We have evaluated and compared both steps of the process. Encapsulation efficiency was assessed by Trypsin wash, which was breaking down particle-trapping proteins and therefore removes all non-internalized particles from cell surface. Total adhesion and encapsulation efficiency was assessed by PBS wash, which preserve particles attached to cell surface. Using an automatic fluorescent microscope system, we have imaged random regions of 386-well flat-bottom plates with seeded and attached cells 1 h after nanoparticle addition. The viability was above 92% determined by live/dead nuclei fluorescent stain for all washing treatments. Morphology of HM and LM human breast cancer cells on the rigid surface is remarkably similar (Fig. 1). The distribution of adhered and encapsulated and encapsulated only fluorescent nanoparticles cannot be distinguished without additional analysis. The particles were distributed mainly near the cell edges for both HM and LM breast cancer cells. Almost no particles were detected on the surface and inside benign cells. No visible difference was detected between cells internalizing particles of size 100 and 200 nm, however almost no internalized particles were observed in benign cells as demonstrated in magnification on Fig. 1 and with lower magnification in Supplementary 1.

**Figure 2:**
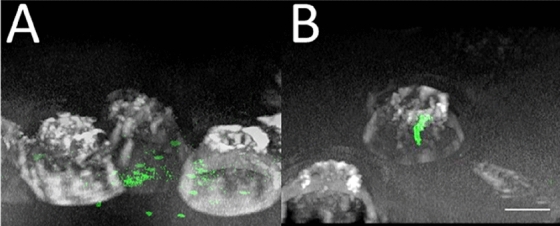
3D reconstruction images of adhered and internalized (**A**) and internalized only (**B**) carboxylate modified 200 nm nanoparticles by high MP breast cancer cells. The scale bar is 10 µm.

Pseudo confocal imaging with a Z-stack step of 1–2 µm, was performed for each cancer cell type and treatment type to prove the presence of fluorescent nanoparticles within the imaged cells. As shown in Fig. 2, the nanoparticles are present the all-cell depth and not only on the cell surface. Moreover, after trypsin washes, the particles were mainly detected inside the cells (internalized particles only), while after PBS wash, a sufficient particle amount was detected on the cell surface (internalized and adhered particles).

Carboxylate-modified polystyrene nanoparticles (of nominal sizes of 100 nm and 200 nm) loaded with fluorescent dyes to create intense fluorescence, typically show little or no photobleaching, even when excited with the intense illumination required for fluorescence microscopy. Due to their wide availability to the scientific community, the particles were used as model particles in to understand the internalization and adhesion in epithelial cell lines. The analysis of fluorescent nanoparticles colocalization with fluorescently labeled cell nuclei was performed and scatter-plotted (Supplementary 3). We have calculated Pearson regression coefficient, R linear overlap coefficient, slope, and intercept of scattering, and have chosen to use Pearson’s correlation coefficient to measure the statistical relationship, or association, between the two variables. The coefficient is known as the best method of measuring the association between variables of interest because it is based on the method of normalized covariance; it gives information about the magnitude of the association, or correlation, as well as the direction of the relationship^37^. We have calculated Pearson coefficients for at least 110 FOVs per condition, resulting in more than 1000 imaged cells from each type and treatment. The distribution of Pearson coefficients for all cell types and treatment types was normal-like (Supplementary 4). In order to confirm the actuality of Pearson coefficient usage for quantitative cross correlation, the several obtained fluorescent images have been divided into 100 equal sections (Supplementary 5A and B). The intensity of TRITC green fluorescence signal (particles) and DAPI blue fluorescence signal (nuclei) was recorded separately for each section (Supplementary 5C). The high correlation of the signals (high TRITC signal with high DAPI signal, as shown on Supplementary 5 inset) 0.6–0.8 was recorded for all checked HMP cell images, and low correlation 0.2–0.4 for LMP cell images. It confirms the results obtained with semi-automated calculation of Pearson coefficients, however this method of analysis is time consuming and hardly automatized, therefore cannot be applied to all our data.

**Figure 3:**
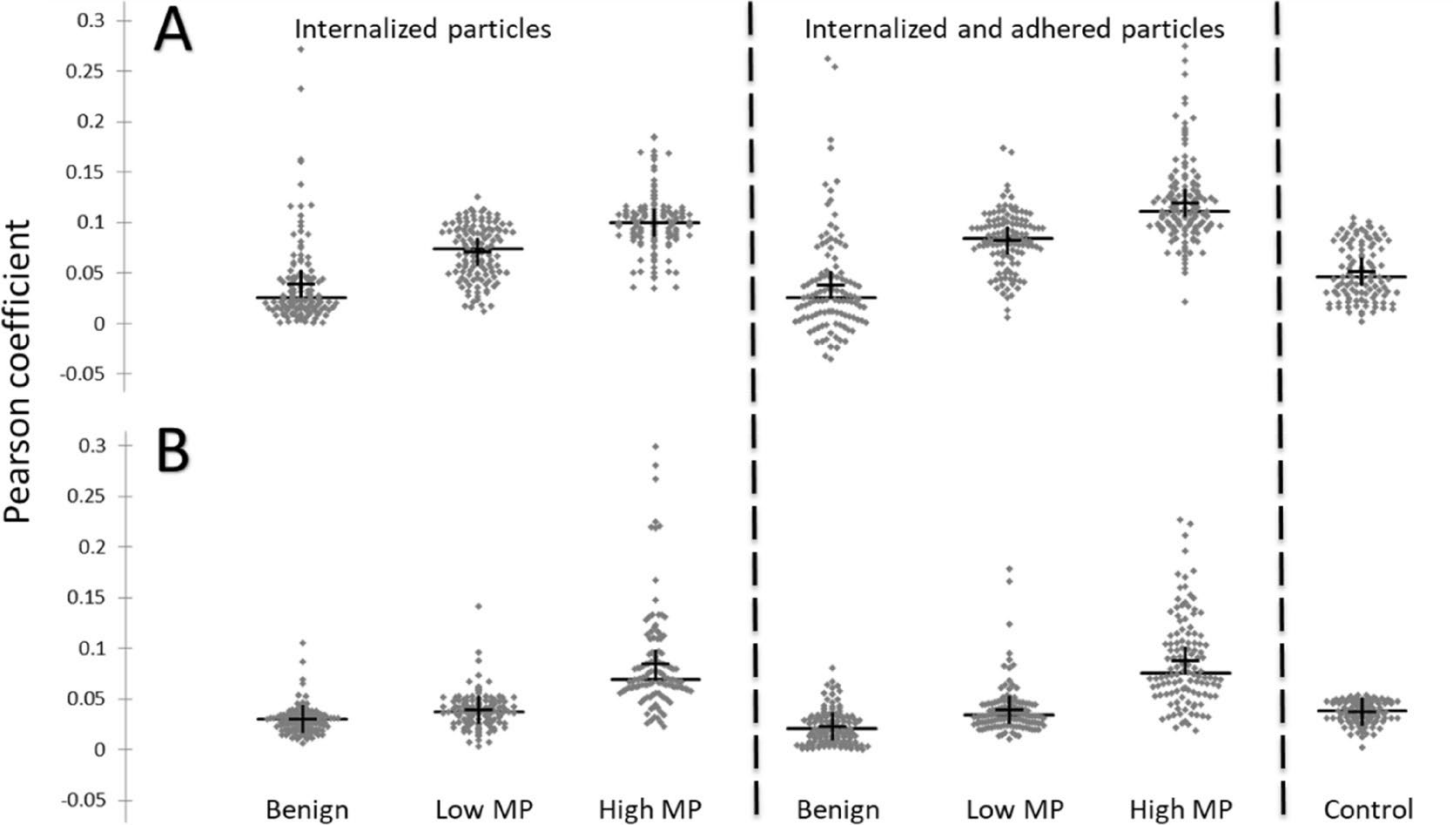
Scattergrams of Pearson coefficients for (**A**) 200 nm and (**B**) 100 nm diameter nanoparticles colocalization with breast cancer cells with high and low MP (MDA-MB-231 and MCF7, respectively) and benign cells (MCF10-A). Mean value is marked by + , median by − . Statistical significance for (**A**) and (**B**) is shown in Tables 1 and 2 respectively.

The obtained results show a clear and significant difference (*P* < 0.0001) between HM cells, LM cells, and benign cells for both tested particle sizes (Fig. 3, Tables 1, and 2). For a short time (1 h), HM cells can internalize 2.18 times and 1.4 times more 100 and 200 nm nanoparticles, respectively, than LM cells. Simultaneously, HM cells internalize and adhere 2.22 times and 1.46 times more 100 and 200 nm nanoparticles, respectively, than LM cells, making the addition of adhered particles negligible for detecting the difference between cells with various metastatic potentials. Moreover, HM cells encapsulated 2.85 times and 2.56 times more 100 and 200 nm nanoparticles respectively than benign cells. Despite LM cells encapsulating only 1.3 times and 1.85 times more 100 and 200 nm nanoparticles, respectively, than benign cells, it still makes the differentiation of metastatic cells from benign cells feasible. Thus, the nanoparticles internalization together with adhesion allows differentiating LM from benign cells even clearly (1.74 and 2.15 times more 100 and 200 nm nanoparticles, respectively), while internalization together with adhesion of 100 and 200 nm nanoparticles provided differentiation between HM and benign cells 3.86 times and 3.15 times, respectively.

**Table 1:** Statistical significance (*P* value) for Pearson coefficients between various cell lines and treatments obtained with 100 nm fluorospheres.

	MCF10A trypsin	MCF7 trypsin	MDA231 trypsin	MCF10A PBS	MCF7 PBS	MDA231 PBS	Control
MCF10A trypsin	–	*P* < 0.0001	*P* < 0.0001	*P* < 0.001	*P* < 0.0001	*P* < 0.0001	*P* < 0.001
MCF7 trypsin	*P* < 0.0001	–	*P* < 0.0001	*P* < 0.0001	*P* = 0.8515	*P* < 0.0001	*P* = 0.2918
MDA231 trypsin	*P* < 0.0001	*P* < 0.0001	–	*P* < 0.0001	*P* < 0.0001	*P* = 0.6967	*P* < 0.0001
MCF10A PBS	*P* < 0.001	*P* < 0.0001	*P* < 0.0001	–	*P* < 0.0001	*P* < 0.0001	*P* < 0.0001
MCF7 PBS	*P* < 0.0001	*P* = 0.8515	*P* < 0.0001	*P* < 0.0001	–	*P* < 0.0001	*P* < 0.0001
MDA231 PBS	*P* < 0.0001	*P* < 0.0001	*P* = 0.6967	*P* < 0.0001	*P* < 0.0001	–	*P* < 0.0001
Control	*P* < 0.001	*P* = 0.2918	*P* < 0.0001	*P* < 0.0001	*P* < 0.0001	*P* < 0.0001	–

**Table 2:** Statistical significance (*P* value) for Pearson coefficients between various cell lines and treatments obtained with 200 nm fluorospheres.

	MCF10A trypsin	MCF7 trypsin	MDA231 trypsin	MCF10A PBS	MCF7 PBS	MDA231 PBS	Control
MCF10A trypsin	–	*P* < 0.0001	*P* < 0.0001	*P* = 0.8535	*P* < 0.0001	*P* < 0.0001	*P* < 0.01
MCF7 trypsin	*P* < 0.0001	–	*P* < 0.0001	*P* < 0.0001	*P* < 0.005	*P* < 0.0001	*P* < 0.0001
MDA231 trypsin	*P* < 0.0001	*P* < 0.0001	–	*P* < 0.0001	*P* < 0.0001	*P* < 0.0001	*P* < 0.0001
MCF10A PBS	*P* = 0.8535	*P* < 0.0001	*P* < 0.0001	–	*P* < 0.0001	*P* < 0.0001	*P* < 0.02
MCF7 PBS	*P* < 0.0001	*P* < 0.005	*P* < 0.0001	*P* < 0.0001	–	*P* < 0.0001	*P* < 0.0001
MDA231 PBS	*P* < 0.0001	*P* < 0.0001	*P* < 0.0001	*P* < 0.0001	*P* < 0.0001	–	*P* < 0.0001
Control	*P* < 0.01	*P* < 0.0001	*P* < 0.0001	*P* < 0.02	*P* < 0.0001	*P* < 0.0001	–

Encapsulation and adhesion efficiency of benign cells are the same order of the recorded control background (negative and positive controls obtained for cells without particles and particles without cells are not differ statistically and therefore have been combined to one control group for each particle size Fig. 3). It means that normal (benign) cells do not internalize or adhere to 100 and 200 nm particles during the measured time period of 1 h. We can see (Table 1) that with 100 nm particles, there is no significant difference between washed by trypsin and by PBS breast cancer cells, both LM and HM. It means that adhering efficiency of 100 nm nanoparticles by cancer cells is very low, and all non-internalized particles have been washed out from the cell surface. In contrast, the obtained for 200 nm particles colocalization coefficients are significantly higher (Table 2) after PBS wash (compared to trypsin wash) for both HM and LM cells. It means that, in addition to internalizing nanoparticles, breast cancer cells also firmly adhere the 200 nm particles to their surface.

**Figure 4:**
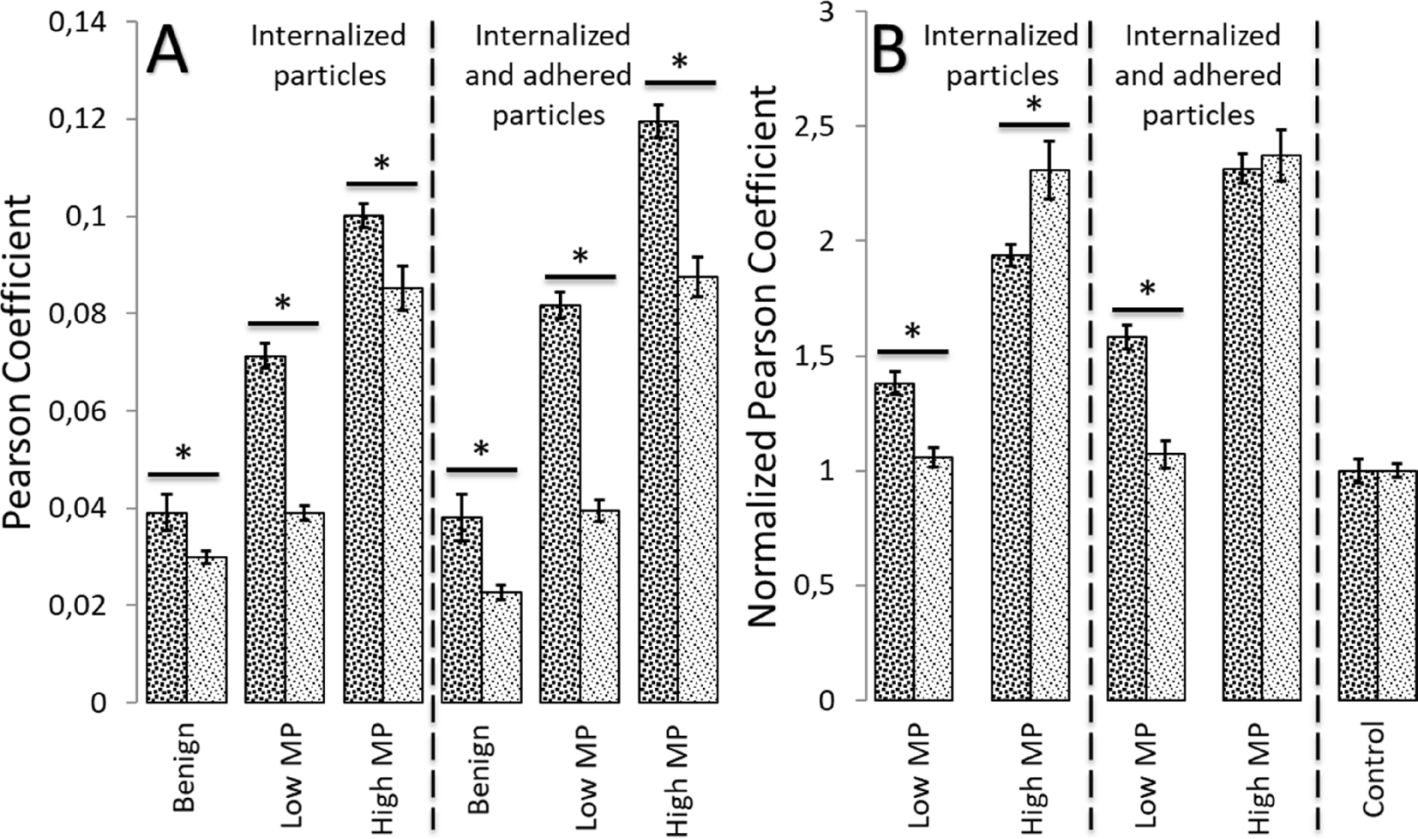
(**A**) Pearson coefficients obtained with 100 (light gray) and 200 (dark gray) nm particles for all tested cell types; n > 110. (**B**) Normalized to control background values of Pearson coefficients obtained with 100 (light gray) and 200 (dark gray) nm particles for high and low MP breast cancer cells. Error bars are standard errors; * − *P* < 0.0001.

The overall colocalization of 200 nm particles in comparison to 100 nm particles (Fig. 4A) is significantly higher (*P* < 0.001) for all tested cell types, both for internalized only and internalized and adhered particles. The Pearson coefficients obtained for HM cells incubated with 200 nm particles at 4C were 0.03 ± 0.007 and 0.025 ± 0.0041 for internalized and adhered and internalized only particles, respectively. The obtained values are the same order, as values obtained for the benign cells at 37C, and more than 3 folds lower than values for HM cells at 37C (Fig. 4A). The fact, that coefficients obtained for HM cells at 4C are not significantly different from the control background, confirm that the particles internalization is driven by endocytosis mechanisms. Normalizing the obtained coefficients to the control background level (separately to each particle size) allows us to see that the coefficients for HM cells are almost 2.5 times higher than the background level for 100 nm particles; however, the coefficients for LM cells are not significantly different from background of 100 nm particles (Fig. 4B). In contrast, the internalization and adhesion of 200 nm particles by LM cells are 1.5 times higher than the background, and by HM cells are more than 2 times higher. Hence, the usage of 200 nm particles seemed preferable because it not only distinguishes metastatic and benign cells but also differentiates between cells with low and high MP.

**Figure 5:**
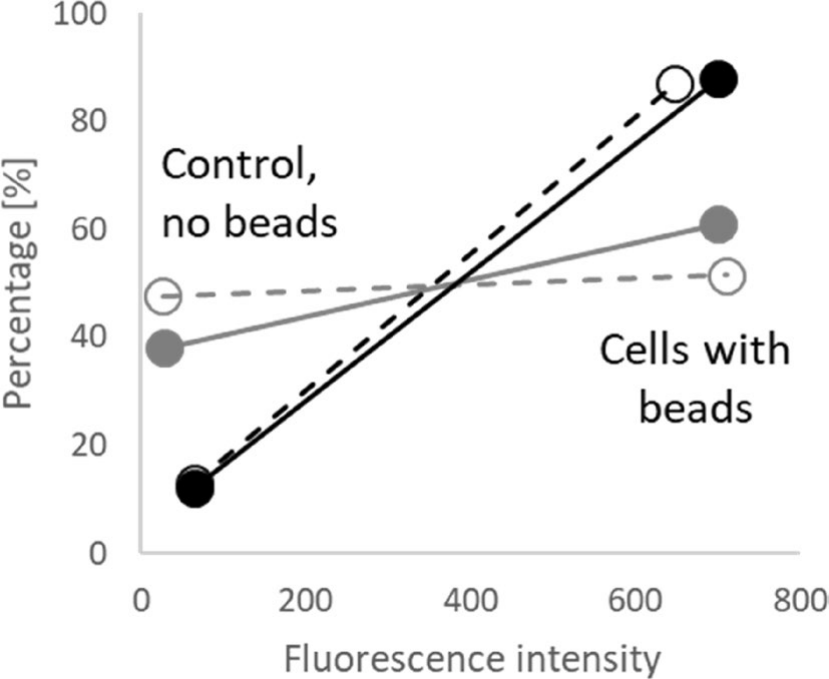
Results of flow cytometry analysis of HM (black) and LM (gray) cells with internalized (dotted line) and internalized and adhered (solid line) 200 nm fluorescent beads.

Conventional flow cytometry measures and analyzes particles between 1 and 15 microns in diameter, although the detection of nanoparticles (NPs of 0.1–0.2 microns) requires specialized systems^38^. Unfortunately, there are limitations in sensitivity of conventional flow cytometry to characterize NPs. It was shown that 100–240 nm polystyrene NP`s, cannot be characterized by conventional flow cytometry, and required additional high-resolution instruments. Small particles had significantly lower measurable signals due to becoming more diffuse and, in some cases, reaching values equivalent to background levels^39^. Despite the above listed limitations, we have successfully differentiated HM and LM cells incubated with 200 nm particles (87% and 51–60% cells with particles from total for HM and LM cells respectively) using conventional flow cytometry technique (Fig. 5 and Supplementary 6). However, in contrast to imaging, no sufficient difference was obtained between cells with internalized only and internalized plus adhered particles. No reliable results of internalized 100 nm particles were recorded by flow cytometry (data not shown).

## Discussion

The new techniques, independent of genetics, based on mechanical invasiveness of cancer cells (microfluidic, gel indentation assays, migration assays, etc.) demonstrated a high success rate for metastasis detection in research labs^14,40,41^, although due to their methodological complexity, they are still far away from clinical implementation. The detection of endocytosis rate and/or intensity via fluorescent nanoparticles internalized by cells is a promising method due to its propensity for automated microscopy methods. It is still challenging to generalize the current findings due to the variation in the endocytic mechanisms dependent on cell types and different nanoparticles. Different complicated approaches have been used to study cellular endocytosis, including chemical and pharmacological inhibitors, genetic approaches, protein and gene expression levels, specific biomarkers, and different microscopy techniques (fluorescence, electron, and atomic force)^42^.

Cells use endocytosis to regulate signaling receptors and sample the extracellular milieu for appropriate responses. It also affects almost all the steps of metastasis and is a target for the functioning of metastasis suppressors^29^. Nanoparticles have the capacity to interact with the cellular machinery and can enter very different cell types with ease. Therefore, the novel and innovative method based on nanoparticle encapsulation efficacy will have an obvious advantage over conventional invasion/migration assays. Nanoparticles are widely used for targeted drug delivery when particles with specific coatings are encapsulated by cancer cells via endocytosis. The time required for APT-decorated QDs 100 nm particles internalization by NSLC cells, was demonstrated to be as low as 30 minutes^43^. Previously, the enhanced encapsulation of nanoparticles by metastatic cells has been demonstrated for extended periods^17^, however, the encapsulation efficacy has not been directly attributed to metastatic propensity.

Here, we focused the work on carboxylate-modified fluorescent 100 and 200 nm diameter nanoparticles—sizes that being representative of typical naturally circulating ‘‘nanovesicles’’ such as low-density lipoprotein (LDL), very-low-density lipoprotein (VLDL), and others with the diameter around 50–1000 nm, along with larger objects which are, however, still below the limit for specialized phagocytosis^44^. We demonstrated that HM and LM breast cancer cells encapsulated 100 nm nanoparticles and encapsulated and adhered 200 nm nanoparticles during one hour. In contrast, benign cells did not encapsulate and adhere to both 100 and 200 nm particles. As previously shown, the benign cell internalization is slower, and particles only appear to internalize into cells at or near the edge of a growing 2D colony^16^. This is likely due to cell–cell interaction differences between the benign and cancer cells. On the other hand, there was no correlation between changes in the activity of any of the endocytic pathways (measured using an In-Cell Elisa assay) with 3D migration, and adhesion was not detected in NSCLC cells^45^, making our chosen colocalization approach to be very efficient.

The difference in internalization and adhesion nanoparticles of HM and LM cells seems to be due to different mechanisms for particle transport. Both rely on fluctuating microtubules (MTs) and their associated molecular motors for intracellular particle transport, yet differ in the relative importance of each mechanism and participation of the actin; this depends on cell structure and cytoskeleton localization^18^. Previously, it was found that HM cells are internally softer but apply more forces on the surroundings and can migrate deeper and with a higher rate than LM cells^26,46^. Thus, the increased internalization ability observed here due to apparently cell softening and more vital forces likely aid in breast-cell metastasis formation.

The ability of cells to encapsulate 200 nm particles more sufficiently than 100 nm particles might be due to the difference in the endocytosis pathway. The 200 nm particles are preferably encapsulated through Clathrin-mediated endocytosis (CME), while 100 nm particles are more likely internalized through Caveolin-mediated endocytosis (CavME)^34^. Components of CavME have a vital role in cell migration, invasion, and metastasis. It was speculated that CAV-1 has a dual role in cancer progression and metastasis. It functions predominantly as a tumor suppressor in the early stages of the disease, whereas at later stages, its expression is associated with tumor progression and metastasis^29^. Caveolin-1 was found upregulated in tumors characterized by an aggressive, metastatic phenotype, and its expression in prostate cancers correlates with poor prognosis^47^. It was also proposed that nanoparticles, bigger than the diameter of the typical clathrin-coated vesicle, can be internalized by CME through the actin elongation of the clathrin-coated pit^48^. The requirement for actin recruitment can slow the endocytic process, leading to altered internalization kinetics compared with conventional CME^49^. Signaling through CME is critical in cancer and metastasis. Clathrin light-chain isoform (CLCb) is upregulated explicitly in NSCLC cells and is associated with poor prognosis^29^. CME inhibition combined with gefitinib treatment decreased NSCLC cell survival and induced apoptosis, resulting in tumor regression accompanying apoptosis in xenograft mouse models^50^.

Any single pharmacological treatment fails to inhibit completely NP uptake, and this leaves open the possibility that within one cell type, they use multiple pathways simultaneously to internalize the same nanoparticles^44^. It was shown that nanoparticles uptake was most effectively reduced when active processes were inhibited, indicating that cells need to spend energy to internalize nanoparticles. Similarly, the ability of cells to invade 2D synthetic hydrogels was significantly inhibited by the single pharmacological treatment Paclitaxel^51^, which mechanism of action is to hyper-stabilize actin filaments. Comparably to actin cytoskeleton involvement in the endocytosis process, actin cytoskeleton was found to play a key role in the migration of cancer cells through narrow pores^26^.

In summary, we accurately distinguished nanoparticle mass bound to the plasma membrane from internalized fluorescent objects and have shown differences in particle uptake by three closely related breast epithelial cell lines with different metastatic potential, which could significantly impact theragnostic and particle tracking applications. Targeting endocytic machinery could be a viable and promising therapeutic and diagnostic strategy for cancer and metastasis. The method proposed here is straightforward; it does not require expensive materials and equipment or laborious cell manipulations; it can successfully overcome the enhanced heterogeneity of TNBC cells and could potentially be applied to other cell lines. Our method, in contrast to the conventional flow cytometry was able to characterize small particles (< 200 nm). Moreover, to achieve a reliable set of data by conventional flow cytometry, the cell number for each experiment should be increased by three orders of magnitude, what is usually hard to collect from core needle biopsy (CNB) samples from breast cancer patients. Besides, the increase in cell number compulsory leads to increase in nanoparticle number, which makes the method more expensive. Rapid (1 h), quantitative, potentially allowing patient­ specific determination of cancer progression likelihood from biopsy/surgery sample, the method may directly affect the choice of treatment protocols for TNBC patients and result in higher survivability and quality of life. We believe that with further advances in metastasis prognosis strategies, TNBC patients will have the opportunity to achieve better clinical outcomes.

## Methods

### Cell culture

We have used two commercially available human epithelial cell lines from breast cancer with high (MDA-MB-231) and low (MCF7) metastatic potential (from American Tissue Culture Collection (ATCC), Manassas, VA). Cell lines were cultured in their appropriate media, as recommended by ATCC: we used Dulbecco’s modified Eagle’s medium (DMEM) (Thermo Fisher, Gibco, Waltham, MA) supplemented with 10 vol% fetal bovine serum (FBS) (Dia-M, Moscow, Russia), and 1 vol% each of L-glutamine (OOO NPP PanEco, Moscow, Russia), penicillin–streptomycin (OOO NPP PanEco, Moscow, Russia), and sodium pyruvate (OOO NPP PanEco, Moscow, Russia). Benign cells (MCF10-A), used as control, were grown in DMEM/Ham’sF-12 medium supplemented with 5% horse serum (Hyclone, Waltham, MA),1vol.% of l-glutamine and penicillin/streptomycin, 10 μg/ml insulin, 10 ng/ml epidermal growth factor, 0.5 μg/ml hydrocortisone, and 100 ng/ml cholera toxin; experiments were run with FBS containing media. Cells were cultured at 37 C, 5% CO2, and high humidity. No mycoplasma testing was done. Cells were frozen at low passages from ATCC stock (i.e., 3–5), and for experiments, cells were thawed and used in passages 7–30 from the ATCC stock.

### Experimental system

We have used yellow-green (excitation/emission 505/515 nm) carboxylate-modified fluorescent 100 and 200 nm in diameter particles (Molecular probes, Invitrogen life technologies, Carlsbad, CA) to achieve the adhesion and encapsulation efficiency of the cells. The cells were seeded 24 h prior to imaging on 386-well flat-bottom plates (Corning Incorporated, Corning, NY). The cell seeding density was 100,000 cells/ml, allowing to obtain about 1000 closely situated, yet not forming a monolayer, cells in each well (well area 5,6 mm^2^). The fluorescent particles and nuclei stain (NucBlue Live Cell Stain ReadyProbes reagent, Eugene, OR) were added 1 h prior to imaging and incubated at 37C. The final particle concentration of approximately 2000 particles/cell was chosen as optimal based on the comparison of translocation coefficients of fluorescence intensity (Supplementary 2). For adhesion experiments, the cells were extensively washed with phosphate buffered saline (PBS) before the imaging (2 times for 2 min by pipetting at room temperature (RT)). For encapsulation experiments, the cells were washed with PBS (2 min, RT), followed by Trypsin (OOO NPP PanEco, Moscow, Russia) wash (2 min, RT). The viability was validated by live/dead nuclei fluorescent stain (NucBlue Live Cell Stain ReadyProbes reagent, Eugene, OR). In each experiment, positive controls (particles without cells) and negative controls (cells without particles) were conducted using the same conditions. At least four biologically independent experiments for each cell type and condition were performed. To confirm that the particles internalization is driven by endocytosis mechanisms, the control experiments were carried out at 4C (the incubation with nano-particles for 1 h), with all other experimental conditions remains identical to the described above.

### Flow cytometry

The MCF7-A and MDA-MB-231 cells with different particle internalization capacities, pre-incubated with 200 nm fluorescent particles, were quantified by flow cytometry (Bio-rad Inc., Hercules, CA, USA). The same number of particles was added to each plate (about 2000 particles/cell) to allow comparison of amounts of particles internalized between different cell lines. Cells were washed as previously described, to remove any unincorporated particles and then harvested using trypsin–EDTA solution at same time point for all cell-lines following particle addition to the media. The cells were centrifuged and resuspended in PBS with 1% BSA. The relative number of particles inside each cell was estimated through measurement of fluorescence levels, where more particles induce proportionately stronger fluorescence. Measurements included at least 20,000 cells compared to control cells without particles at each particle-exposure time point.

### Imaging

Transmitted light and fluorescence (DAPI and TRITC) cell digital images were obtained using the high-content fluorescence inverted imaging microscope ImageXpress Micro XL System (Molecular Devices LLC, San Jose, CA, USA) equipped with a 40x/0.60 NA air-immersion, a long working distance objective lens. The imaging was performed immediately after cell washing at RT for 15–20 min duration. The cell-seeding with the following washing resulted in an average of 20 ± 10 viable cells per field-of-view (FOV) (area of FOV 0,125 mm^2^). At least 20 wells with 4 FOVs per well were automatically imaged in each experiment. Only FOVs containing more than four cells and not containing image artifacts (like unsuspected focal depth change or overexpression) have been proceeded for further analysis, resulting in at least 110 FOVs per condition. At least 1100 cells were imaged at four independent experiments for each experimental condition. Pseudo confocal imaging with a Z-stack step of 1–2 µm was performed on each cancer cell type and treatment type to ensure the presence of fluorescent nanoparticles within the imaged cells.

### Analysis

Build-in image analysis algorithms (ImageJ, National Institutes of Health, Bethesda, MD, and LOCI, University of Wisconsin, Madison, WI) have been used to build 3-D cell images. Custom image analysis algorithms have been created and utilized to find quantitative colocalization (Pearson’s and Overlap coefficients, scatter slope) of fluorescent nanoparticles with imaged cells. In order to confirm the actuality of Pearson coefficient usage for quantitative cross correlation, the obtained images have been divided to equal 100 sections. The intensity of green fluorescence signal (particles) and blue fluorescence signal (nuclei) was recorded separately for each segment. The cross-correlation coefficient (*CC*) of the two fluorescent signals was calculated for several images, according to the following equation:

$$CC = \frac{n\sum xy - \sum x\sum y}{{\sqrt {[n\sum x^{2} - \left( {\sum x} \right)^{2} ]\left[ {n\sum y^{2} - (\sum y)^{2} } \right]} }}$$

when *x* and *y*—the intensity of fluorescent signals for each section, n—number of Sects. (100 in our case)^52^.

The significance of variations between cell lines or adhesion and encapsulation efficiency was determined using the general, linear mixed model, a multivariate regression method for the analysis of variance (ANOVA), with a *P* < 0.05. Calculations were performed using Excel (MicroSoft, Redmond, WA) or MedCalc2018 (MedCalc Software, Ostend, Belgium); scattergrams and descriptive statistics were performed by XLSTAL (Addinsoft, Paris, France).

## Supplementary Information

Supplementary Information.

## Data Availability

The datasets used and/or analyzed during the current study available from the corresponding author on reasonable request.
