# Supplementary material for: Encapsulation and adhesion of nanoparticles as a potential biomarker for TNBC cells metastatic propensity
Source: Sci Rep. 2023 Jul 29;13:12289. doi: 10.1038/s41598-023-33540-1 (PMC10387085; doi:10.1038/s41598-023-33540-1)
Supplement: Supplementary file 1 — Supplementary Information. [file 41598_2023_33540_MOESM1_ESM.docx]

**Supplementary**

**
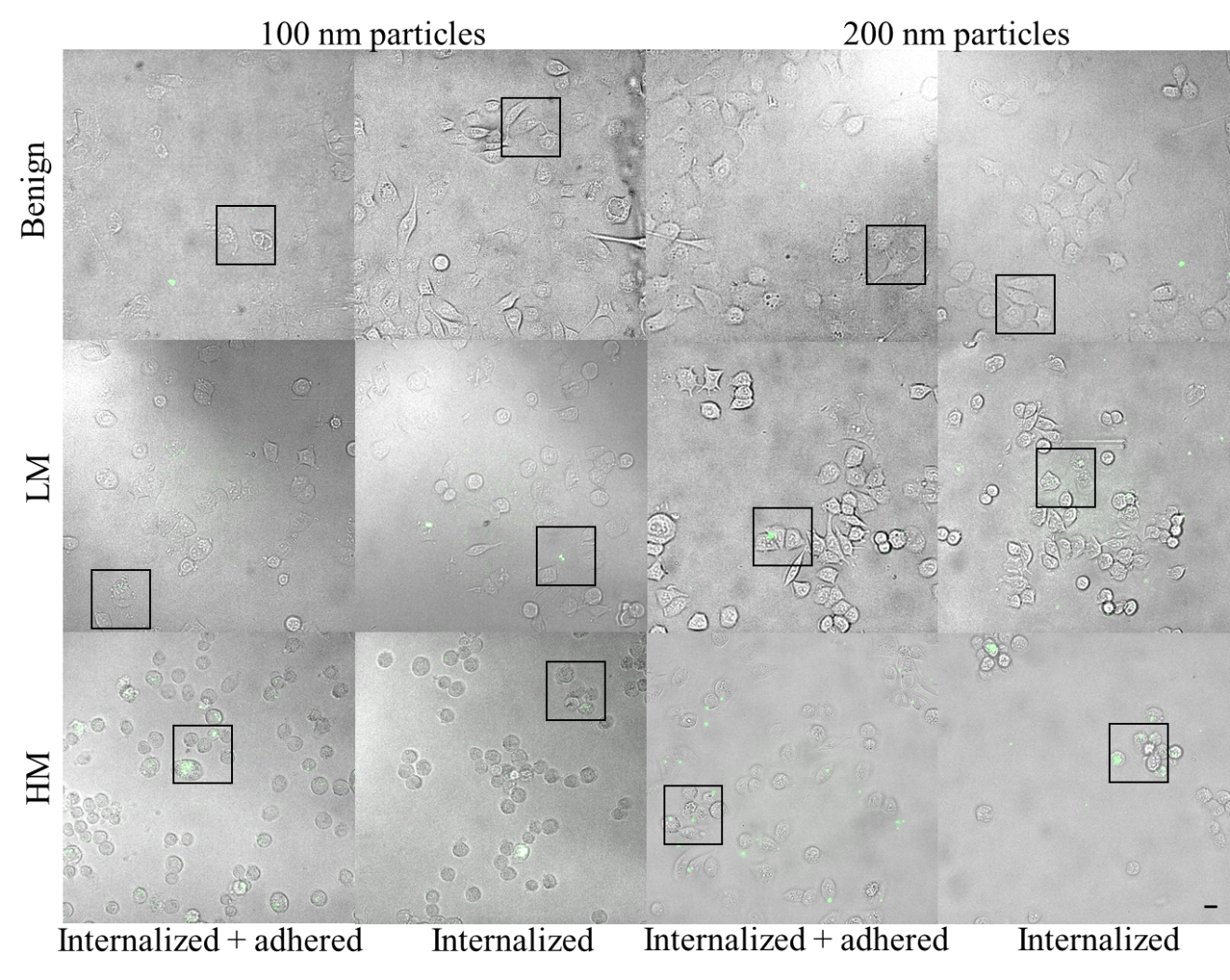
**

Supplementary 1: A typical images of internalized and internalized and adhered carboxylate modified 100 nm and 200 nm nanoparticles by benign cells, cells with low (LM) and high (HM) metastatic potential. HM and LM cells were extensively washed with Trypsin or PBS after 1 h incubation with fluorescent nanoparticles, resulting in the presence of internalized or internalized and adhered beads, respectively. The scale bar is 10 µm. The selected areas (marked by black squares) are presented in magnification 1:5 on Figure 1.

| Number of NP`s/ml calculation | Conc1 [NP’s/cell]  1000 | Conc2 [NP’s/cell]  2000 | Conc3 [NP’s/cell]  3000 |
| --- | --- | --- | --- |
| $N=\frac{6C*{10}^{12}}{\rho*\pi*\phi^{3}}$  C = concentration of suspended beads in g/mL  φ = diameter of microspheres in µm  ρ = density of polymer in g/mL (1.05 for polystyrene) |  |  |  |
|  | Translocation coef.  0.29±0.016 | Translocation coef.  0.37±0.04 | Translocation coef.  0.36±0.06 |
|  | 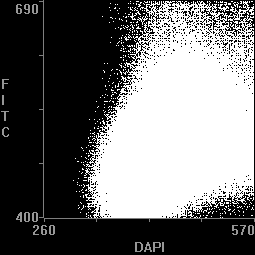 | 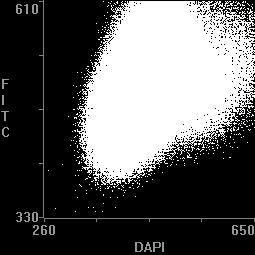 | 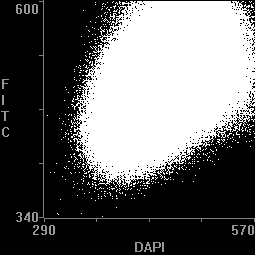 |

Supplementary 2: Nano-particles (NP’s) concentration calculation and concentrations used for the initial optimization of experimental conditions. Translocation coefficient between intensity of green fluorescent particles (FITC) and stained nuclei (DAPI) for conc1 (1000 NP’s/cell) was significantly lower than coefficients for conc2 and conc3 (p<0.05), however conc2 and conc3 are not significantly differ, therefore for the further experiments the concentration of 2000 NP’s/cell have been chosen.


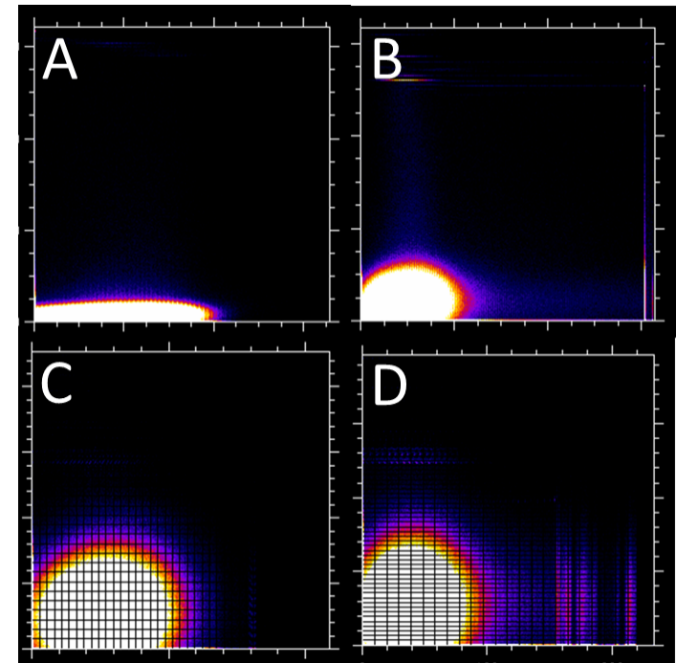


Supplementary 3: Cell-nuclei and 200 nm particles typical co-localization scatter plot, allowing calculating Pearson regression coefficient, R linear overlap coefficient, slope and intercept of co-appearance. (A) and (B) – internalized particles by LM and HM cells respectively; (C) and (D) internalizes and adhered particles by LM and HM cells respectively.

**
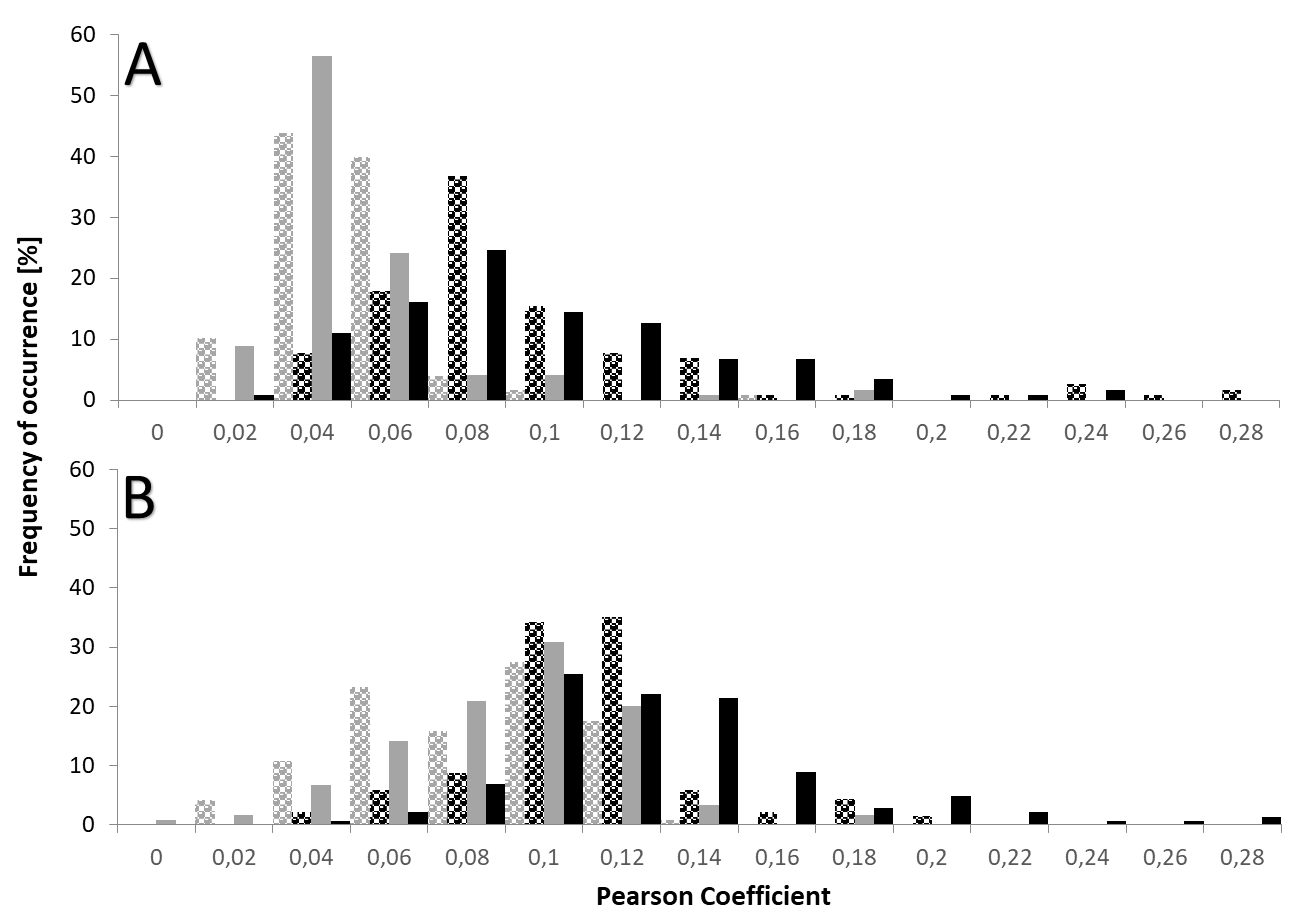
**

Supplementary 4: Distribution histogram of automatically calculated Pearson coefficients for breast cancer cells colocalization with (A) 100 nm and (B) 200 nm particles. Black bars – adhered and internalized particles by HM cells; black doted bars - internalized particles by HM cells; gray bars - adhered and internalized particles by LM cells; gray doted bars - internalized particles by LM cells.


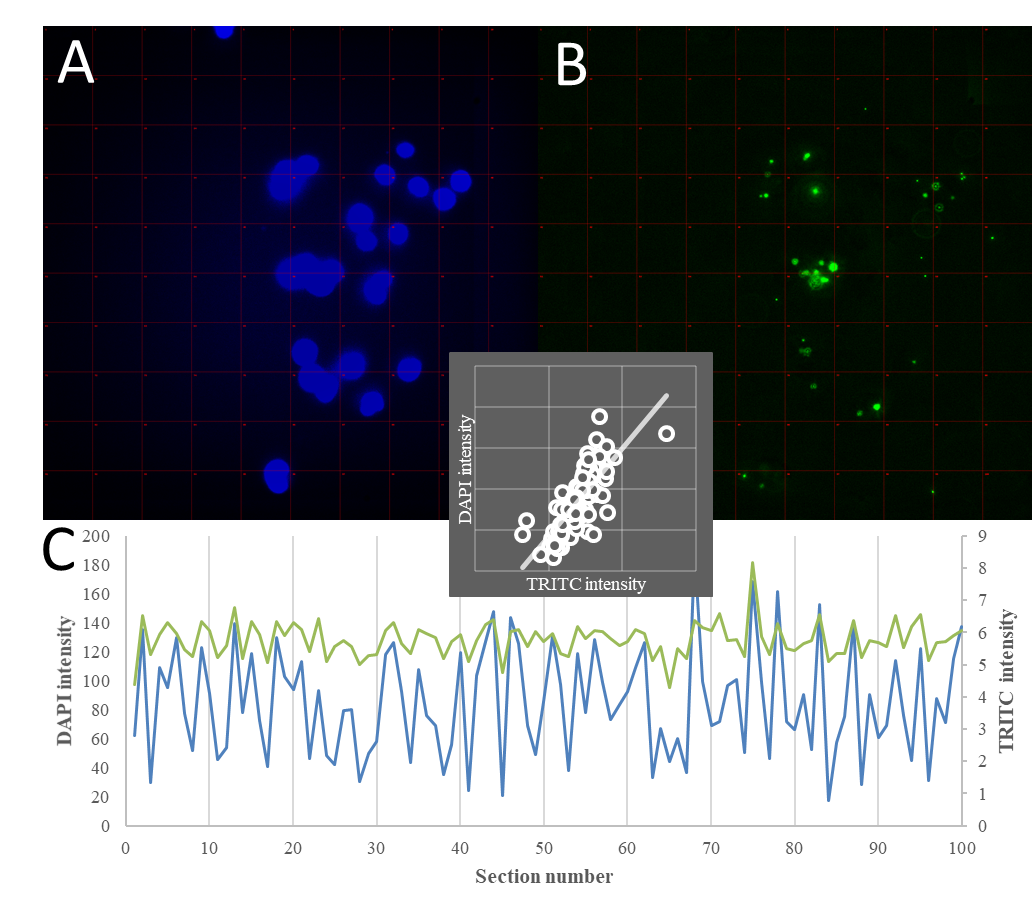


Supplementary 5: The images of DAPI labeled cell nuclei (A) and 200 nm TRITC fluorescent particles (B) divided into 100 equal sections. The fluorescent intensity in either DAPI (blue) or TRITC (green) channel recorded to each section separately (C). The inset shows the correlation of two fluorescent channels.


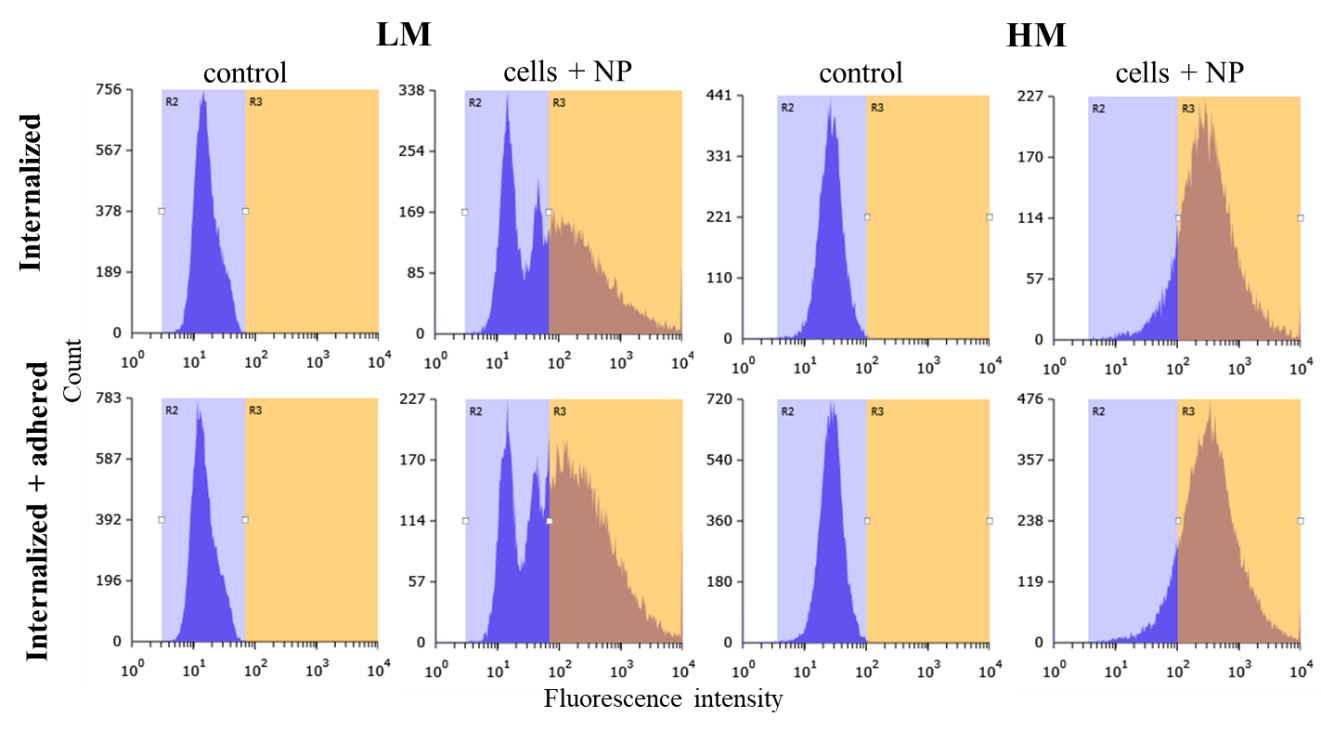


Supplementary 6: Representative histograms of flow cytometry analysis of HM and LM cells with internalized and internalized and adhered 200nm fluorescent particles. R2 region represent cell autofluorescence while R3 represent the fluorescence induced by the nano particles (NP).
